# Psychometric validation of the Paykel Suicidal Behavior Scale in Mexican adolescents

**DOI:** 10.11606/s1518-8787.2026060007086

**Published:** 2026-03-16

**Authors:** Marina Séris-Martínez, Fernando Austria-Corrales, Yendy Cruz Hernández, Berenice Pérez-Amezcua, Alberto Jiménez Tapia, Claudia Iveth Astudillo-García, Leonor Rivera-Rivera

**Affiliations:** I Instituto Nacional de Salud Pública. Centro de Investigación en Salud Poblacional. Cuernavaca, México; II Universidad Autónoma del Estado de Morelos. Centro de Investigación Transdisciplinar en Psicología. Cuernavaca, México; III Instituto Nacional de Psiquiatría Ramón de la Fuente Muñiz. Ciudad de México, México

**Keywords:** Suicide, Adolescent, Education, Primary and Secondary, Factor Analysis, Statistical, Mass Screening

## Abstract

**OBJECTIVE:**

To psychometrically validate the Spanish version of the Paykel Suicidal Behavior Scale in the Mexican adolescent population, and to establish an optimal cut-off point to identify risk of suicidal behavior in school contexts.

**METHODS:**

A cross-sectional study was conducted in 2022 with a non-probabilistic sample of 1,407 students from eight public high schools in the state of Morelos, Mexico. The mean age was 17 years, 58.7% were female and 41.3% were male. The students completed an online questionnaire that included the Paykel Suicidal Behavior Scale, the Center for Epidemiological Studies Depression Scale, and the Depression Anxiety and Stress Scale. A confirmatory factor analysis, item response theory, factorial invariance analysis (by sex, gender identity, and school grade), divergent validity analysis, and ROC curves were applied in this study.

**RESULTS:**

The confirmatory factor analysis was found to be acceptable. The factor loadings ranged from 0.799 to 0.938. Item discrimination parameters were elevated (2.33 to 6.63), with difficulties ranging from 0.17 to 1.11. Factor invariance was confirmed in all subgroups. The divergent validity of the Paykel Suicidal Behavior Scale was satisfactory, as evidenced by its moderate correlations with the Epidemiological Studies Depression Scale (r = 0.507), depression (r = 0.644), anxiety (r = 0.570), and stress (r = 0.541). ROC analysis identified an optimal cutoff point of ≥ 1.0, with sensitivity of 75.93% and specificity of 76.54%.

**CONCLUSION:**

The Mexican version of the Paykel Suicidal Behavior Scale demonstrates robust psychometric properties, including validity, reliability, and factorial invariance in the adolescent school population. The scale’s brevity, clarity, and ease of application make it an effective tool for school screening, allowing for timely detection and referral to mental health services. Its use is recommended in adolescent suicide prevention programs in educational contexts in Mexico.

## INTRODUCTION

 Suicide poses a significant global public health challenge. It is usually a process that encompasses a spectrum of phases, including suicide ideation, planning, suicidal behaviors, and attempts^1-3^. Research indicates that ideation can serve as a predictor of suicide attempts, and these attempts can in turn predict future risk of death by suicide^4^. It is estimated that, for every suicide death, there may be as many as 20 previous attempts^5^. The complexity of this phenomenon highlights the importance of developing effective strategies. These strategies should include cost-effective and psychometrically robust questionnaires to identify early indicators of risk. Furthermore, interventions should be implemented targeting groups with higher prevalence and vulnerability, such as the adolescent population^6,7^. 

 Data obtained through surveys with representative and global samples have revealed that adolescents between 12 and 16 years of age living in middle and low-income countries found the following: 10% reported experiencing suicidal ideation, 10% reported having created a plan for attempting suicide, and 11% reported making an attempt in the past 12 months. The Americas exhibited the highest prevalence^7^. According to the findings of another study, the prevalence of suicidal ideation, planning, and attempts was 19%, 16%, and 9%, respectively, among individuals aged 14 to 18 in the United States. Notably, these figures increased considerably among women between 2019 and 2021, with ideation rising from 24% to 30%, planning increasing from 20% to 24%, and attempts rising from 11% to 13%^8,9^. In Latin America, significant variations are shown in the prevalence of ideation (12%–23%), planning (7%–23%), and attempt (8%–21%), which are higher in women than in men^10^. 

 In Mexico, the prevalence of ideation has been reported at 8%, while the prevalence of attempted suicide is 7%. However, when asked about attempts in the past 12 months, the figure was 3%, with higher values for females^1,11^. The existing data indicate that the adolescent population reports high rates of suicide ideation, planning, and attempts, which places them at increased risk of suicide. Given the typical challenges associated with this stage, such as emotional regulation difficulties, impulsivity, social skills limitations, and the presence of depression, anxiety, substance use, and stress^6,7,12^, the importance of reliable and valid instruments for measuring suicide phases becomes evident. A variety of scales and questionnaires have been used with the adolescent population^11,13-15^. 

 However, it should be noted that the tools used to assess the process of suicide are not designed to encompass its different phases. Some of these tools are designed to measure a single aspect of the process, while others assess multiple stages, but are more extensive. For instance, Beck’s suicidal ideation scale, which has been validated in Mexican adolescent students, contains 19 items^16^. The Roberts’ suicidal ideation scale is comprised of four items and has been evaluated among Mexican adolescents, but presents several inconsistencies that may affect its use^17,18^. These assessments primarily address ideation. On the other hand, Plutchik’s suicide risk scale is a 26-item tool designed to assess various factors associated with suicide risk^19^. These factors include previous attempts, the intensity of current ideation, depression, and hopelessness. Therefore, the Paykel Suicide Scale (PSS) is a reliable, brief, and user-friendly instrument for adolescents that is also applicable in educational contexts^12,14^. 

### Paykel Suicide Scale

 The PSS was developed in 1974 in the United Kingdom to measure suicide thoughts in psychiatric patients due to the limited availability of information on suicide in the general population at that time^20^. It was subsequently translated into Spanish and culturally adapted in 2018 with Spanish adolescents^15^. The five-question instrument is divided into three dimensions: thoughts of death, suicidal ideation, and suicide attempt. The initial survey contained four response options (always, sometimes, almost never, and never)^20^. In the Spanish adaptation, the response options were made dichotomous^15^. 

 The PSS has been used to assess suicidal behavior (SB) in the adolescent population in some Latin American countries^12-14^. However, it has not yet been validated in the Mexican population. It is also essential to have valid and reliable instruments for the adolescent population, developed with approaches that transcend Classical Test Theory. These approaches should provide substantial evidence regarding the validity of the theoretical construct, the individual behavior of the items, the invariance of the model between subgroups, and the usefulness of establishing cut-off points that allow for the identification of adolescents at risk. 

 The objective of this study is to assess the psychometric properties of the scale in a sample of adolescent students from the state of Morelos, Mexico, and to determine a cutoff value for timely referral to mental health services in case of detecting SB risk. 

## METHODS

### Population and Study Design

 We used a secondary database of a cross-sectional survey of students from eight high schools in the State of Morelos in 2022. The sample was obtained by convenience and included 1,407 students (sex at birth: 58.7% female and 41.3% male). Of the participants, 52, 39, and 9% identified as female, male, or another gender identity, respectively. The mean age was 17 years (range: 15–22), 40% of the participants were in their first year of high school, 29% in their second year, and 31% in their third year. The participants completed an electronic questionnaire during class time. 

 The teaching staff provided the electronic link that enabled access to the questionnaire. Students who consented to participate and had parental consent completed the questionnaire using computers, cell phones, or tablets. The project was evaluated and approved by the Ethics Committee of the National Institute of Public Health (Registration number: 17CEI00420160708). 

### Variables

#### Suicidal behavior

 The validated Spanish version of the PSS was used to assess SB, specifically thoughts of death, suicidal ideation, and suicide attempts^12,15^. The questionnaire consists of five items:

- *Have you ever felt that life is not worth living?*
- *Have you ever wished you were dead?*
- *Have you considered ending your life, even though you were not actually going to do it?*
- *Have you ever reached the point where you really considered taking your own life or made plans about how you would do it?*
- *Have you ever attempted to take your own life?*

Each question has dichotomous response options Yes and No, which scores 1 and 0, respectively^21^. The dichotomous response format was retained to preserve the original conceptual structure of the PSS as a brief screening tool for the presence or absence of suicidal thoughts or behaviors rather than their frequency or severity. This decision also aimed to facilitate comprehension and reduce cognitive load among adolescents while maintaining adequate psychometric discrimination in previous validations^12,15,17^. 

#### Depression, anxiety, and stress

 The Depression Anxiety and Stress Scale (DASS-21) was used in its short form^22,23^. This scale is composed of three dimensions: depression, anxiety, and stress. The DASS-21 instrument comprises 21 items, with seven items per dimension, and four response options ranging from 0 to 3. A score of 0 indicates "it has not happened to me", a score of 1 indicates "it has happened to me a little", a score of 2 indicates "it has happened to me quite a lot", and a score of 3 indicates "it has happened to me a lot". The content of the items refers to symptoms associated with each of the dimensions^24^. The cut-off points considered for each dimension were as follows: depression (0–4: no depression, 5–6 mild, 7–10 moderate, 11–13 severe, > 14 extremely severe), anxiety (0–3: no anxiety, 4 mild, 5–7 moderate, 8–9 severe, > 10 extremely severe), and stress (0–7: no stress, 8–9 mild, 10–12 moderate, 13–16 severe, > 17 extremely severe)^22,23^. 

#### Depressive symptomatology

 The depressive symptomatology variable was constructed from the abbreviated version of the Center for Epidemiological Studies Depression Scale (CESD-7)^25^. The cut-off value for the presence of depressive symptomatology was determined to be 9 or more points as a global score on the scale. 

 The CESD-7 scale was included in addition to the DASS-21 because both serve complementary purposes: the CESD-7 and DASS-21 were used to explore divergent validity across related affective dimensions, while the DASS-21 depression subscale provided a binary external criterion to identify participants with depressive symptomatology for Receiver Operating Characteristic (ROC) curve analysis. 

### Other Variables

#### Sex at birth

 The sex at birth variable was based on the following question: "*What was your assigned sex at birth?*" The question had two response options: "female" or "male." 

#### Gender identity

 This variable was constructed from the following question: "*How would you best describe your gender identity?*" The following response options were provided: "woman", "man", "transsexual woman", "transsexual man", "gender fluid", "non-binary", "other", or "I don’t know". This variable was recategorized into three categories based on the response frequency ("female", "male", and "other"). 

### Data Analysis

 A confirmatory factor analysis (CFA) was performed using the maximum likelihood estimation method with robust estimation of standard errors, mean, and variance adjusted to assess the latent structure of the SB construct^26^. Standardized factor loadings and item significance were examined. Additionally, an Item Analysis under the 2-Parameter Logistic Model (2PL) was conducted to analyze the psychometric properties of the items through item response theory (IRT), and to estimate the discrimination and difficulty parameters of the items^27,28^. Standard errors associated with the parameter estimates were examined to assess the stability of the model and identify potential overfitting in items with extreme discrimination values. Additionally, item fit statistics and information functions were reviewed to confirm that parameter estimates were consistent with the empirical data. An evaluation of the factorial invariance was performed through four levels: configural, metric, scalar, and strict. The fit indices (Comparative Fit index — CFI, Tucker-Lewis Index — TLI, Root Mean Square Error of Approximation — RMSEA, and Standardized Root Mean Square Residual — SRMR) were compared to assess whether the factorial structure of the instrument remained constant across the different groups^29^. 

 The divergent validity of the instrument was also explored by assessing the relationship between SB with theoretically distinct constructs such as depression. To this end, the CESD-7 scale and the three subscales of the DASS-21 were used: depression, anxiety, and stress. This divergent validity was analyzed using latent correlations between factors, with the expectation of moderate or low coefficients, indicating conceptual independence. 

 To establish an optimal cut-off point to identify students at risk of SB, a ROC curve analysis was performed. The association between the PSS and the depression subscale of the DASS-21-depression was used as a reference, with depression categorized into five levels (0–4 = "no depression", 5–6 = "mild depression", 7–10 = "moderate depression", 11–13 = "severe depression", ≥ 14 = "extremely severe depression"). To facilitate interpretation, the depression variable was dichotomized into two categories: "at risk" (severe or extremely severe) and "not at risk" (mild, moderate, or no depression). The discriminative performance of the PSS was quantified using the area under the curve to evaluate classification accuracy. It was hypothesized that the correlation with the PSS would adequately reflect the risk of SB. 

 The analyses were performed in R software with the packages *lavaan* for the AFC and *mirt* for the 2PL model of TRI. A cut-off value close to 0.95 for the CFI, a cut-off value close to 0.06 for the RMSEA, and a cut-off value close to 0.08 for the SRMR were considered to result in lower Type II error rates (with acceptable Type I error costs). In this context, a suboptimal model fit is defined as a CFI value above 0.90, an SRMR value below 0.10, and an RMSEA value between 0.08 and 0.10. Conversely, an adequate model fit is defined as a CFI value between 0.92 and 0.95, an SRMR value between 0.08 and 0.10, and an RMSEA value below 0.08. Finally, a good model fit is defined as a CFI value above 0.92, an SRMR value below 0.08, and an RMSEA value below 0.05. Finally, measurement invariance between groups in each model was determined when Δχ^2^ p > 0.05, ΔCFI < 0.01, ΔRMSEA < 0.015, and ΔSRMR < 0.030 (for metric invariance) or ΔSRMR < 0.015 (for scalar and residual invariance)^29,30^. 

## RESULTS

### Confirmatory Factor Analysis

 The results of the CFA indicate that the proposed factor structure was adequate, with standardized factor loadings ranging between 0.799 and 0.938. The CFI values were close to 0.95, and the RMSEA values were within the acceptable range (< 0.08) (Table 1). This suggests that the items adequately represent the latent construct of suicide. 

**Table 1:** Factor loadings, discrimination parameters and item difficulty in confirmatory factor analysis and the two-parameter model of item response theory.

	Parameter	Standard Error	p-value	Standardized factorial load	Discrimination (A)	Difficulty (B)	Dimension
F1	1.000	-	-	0.799	2.33	0.25	Thoughts of death
F2	1.113	0.033	< 0.001	0.890	3.59	0.17	Thoughts of death
F3	1.127	0.033	< 0.001	0.900	3.85	0.25	Suicidal ideation
F4	1.174	0.034	< 0.001	0.938	6.63	0.79	Suicidal ideation
F5	1.050	0.037	< 0.001	0.839	4.25	1.11	Suicide attempt

Note: the parameters obtained in the confirmatory factor analysis (CFA) and in the two-parameter model (2PL) of the Item Response Theory (IRT) are presented. The standardized factor loading represents the relationship of each item with the latent factor. Discrimination (A) indicates the ability of the item to differentiate between levels of SB risk, while difficulty (B) reflects the level of risk required to respond affirmatively to the item.

Items: (F1) Have you ever felt that life is not worth living? (F2) Have you ever wished you were dead? (F3) Have you ever considered ending your life, even though you were not actually going to do it? (F4) Have you ever reached the point where you really considered taking your life or made plans about how you would do it? (F5) Have you attempted to take your own life?

### Item Analysis under the Two-Parameter Logistic Model

 In the IRT analysis under the 2PL model, discrimination parameters ranged from 2.33 (F1) to 6.63 (F4), indicating high item sensitivity across the latent trait. Difficulty parameters varied between 0.17 (F2) and 1.11 (F5), consistent with an ordered continuum from lower to higher severity of suicidal behavior. Inspection of standard errors confirmed parameter stability, and no item showed empirical misfit (Table 1). 

### Invariance by Gender Identity, Sex and Grade Level

 Regarding gender identity (male, female, and other), the results suggest that configural, metric, scalar, and strict invariance is maintained, since the RMSEA, CFI, and TLI values are within acceptable ranges. The CFI and TLI values were greater than 0.99 at all levels of invariance, indicating a good model fit. The results for grade level demonstrated evidence of factorial invariance at all levels, with RMSEA values less than 0.07 and CFI and TLI values greater than 0.99. The analysis of invariance by sex also supports factorial equivalence at all levels, with fit indicators falling within the recommended criteria (Table 2). 

**Table 2:** Fit indices of factorial invariance analysis in different groups: gender identity, school grade and sex.

Model	ChiSq	DF	p-value	RMSEA	CFI	TLI	SRMR	Δp_value	ΔCFI	ΔRMSEA	ΔSRMR
Gender identity											
Configural	47.42	15	0.00003	0.069	0.995	0.990	0.041	-	-	-	-
Metric	51.18	23	0.00064	0.052	0.996	0.995	0.048	0.001	0.001	-0.017	0.007
Scalar	51.72	21	0.00021	0.057	0.995	0.994	0.042	-0.000	-0.001	0.005	-0.006
Strict	51.72	21	0.00021	0.057	0.995	0.994	0.042	0.000	0.000	0.000	0.000
Grade level											
Configural	42.90	15	0.00016	0.064	0.996	0.993	0.034	-	-	-	-
Metric	36.13	23	0.03999	0.035	0.998	0.998	0.037	0.040	0.002	-0.029	0.003
Scalar	47.27	21	0.00086	0.052	0.997	0.995	0.034	-0.039	-0.001	0.017	-0.003
Strict	47.27	21	0.00086	0.052	0.997	0.995	0.034	0.000	0.000	0.000	0.000
Sex											
Configural	46.70	10	0.00000	0.073	0.995	0.990	0.038	-	-	-	-
Metric	43.77	14	0.00006	0.056	0.996	0.994	0.043	0.000	0.001	-0.017	0.005
Scalar	48.57	13	0.00001	0.063	0.995	0.992	0.039	-0.000	-0.001	0.007	-0.004
Strict	48.57	13	0.00001	0.063	0.995	0.992	0.039	0.000	0.000	0.000	0.000

Note: the results of the factorial invariance analysis in different groups (gender, school grade, and sex) are presented. The models evaluated include configural invariance (equality in factorial structure between groups), metric invariance (equality in factor loadings), scalar invariance (equality in loadings and intercepts), and strict invariance (equality in loadings, intercepts, and residuals). CFI (Comparative Fit Index) and TLI (Tucker-Lewis Index) values close to 1.0 indicate a good fit, while RMSEA (Root Mean Square Error of Approximation) and SRMR (Standardized Root Mean Square Residual) values less than 0.08 suggest an adequate representation of the model. Significant differences are considered when ΔCFI > 0.01, ΔRMSEA > 0.015, and ΔSRMR > 0.030.

### Divergent Validity

 To evaluate the divergent validity of the PSS, we calculated correlations between the PSS and scales theoretically related to emotional distress. These scales measure different constructs, such as the CESD-7 and the DASS-21. Both of these have been widely used in mental health studies^31,32^. 

 High correlations were observed between the subscales of DASS-21, with values ranging from 0.83 to 0.85. These findings reflect the high interrelationship between these constructs. The PSS scale demonstrates a moderate correlation with the CESD-7 (r = 0.507) and with the depression subscale of the DASS-21 (r = 0.644), indicating a notable relationship between depressive symptomatology and SB. However, these correlations are not sufficiently high to indicate that both scales measure exactly the same construct, providing evidence of divergent validity. The PSS demonstrated a weaker correlation with the stress (r = 0.541) and anxiety (r = 0.570) scales, confirming that, although the SB is associated with emotional symptoms, it measures a distinct and specific construct. These results suggest that the SB scale captures unique information related to suicidal ideation and attempt, beyond the simple presence of depressive or anxious symptoms (Table 3). 

**Table 3:** Correlation matrix between the Paykel scale, Center for Epidemiological Studies Depression Scale, and the Depression, Anxiety and Stress Scale (DASS-21).

Variable	CESD7	DASS_DEP	DASS_ANX	DASS_STRS	PAYKEL
CESD7	1	0.756	0.681	0.739	0.507
DASS_DEP	0.756	1	0.830	0.852	0.644
DASS_ANX	0.681	0.830	1	0.852	0.570
DASS_STRS	0.739	0.852	0.852	1	0.541
Paykel^a^	0.507	0.644	0.570	0.541	1

PSS: Paykel Suicidal Behavior Scale.

Note: correlation coefficients between the PSS scale and other psychological measures are presented: CESD-7: Center for Epidemiological Studies Depression Scale; DASS-DEP (DASS-21: depression subscale), DASS-ANS (DASS-21: anxiety subscale) and DASS-EST (DASS-21: stress subscale).

^a^ The Paykel variable consists of the sum of the questions. This implies that higher values suggest higher risk as well.

### Cut-off Score

 To determine an optimal threshold for identifying adolescents at risk of SB, a logistic regression analysis was performed using the PSS as the predictor variable and the presence of severe or extremely severe depression (according to the depression subscale of the DASS-21) as the criterion variable. The results of the logistic regression model indicated that the PSS is a significant predictor of SB (χ^2^ = 215.28, p < 0.0001), with a considerable positive effect on the probability of presenting risk of such behavior. The model constant was -0.28 (p = 0.0004), while the coefficient estimate for the PSS was 0.99, suggesting that an increase in PSS score significantly increases the probability of SB. 

 Subsequently, a ROC curve analysis was performed to evaluate the PSS’s classification accuracy. The analysis yielded an area under the curve of 0.80591, indicating good discriminative ability. A cut-off point of 1.0 optimized the balance between sensitivity and specificity, achieving 75.9% sensitivity and 76.5% specificity. This threshold effectively identifies approximately three out of four adolescents at risk of suicidal behavior while maintaining an acceptable false-positive rate (Table 4). 

**Table 4:** Sensitivity, specificity and classification of cases at different cut-off points of the Paykel scale.

Cut-off point	Probability	1-Especificity	Sensitivity	Sens(1-Espec)	True positive	True negative	False positive	False negative
5	0.991	0.014	0.148	0.134	143	433	6	825
4	0.976	0.023	0.287	0.264	278	429	10	690
3	0.937	0.036	0.434	0.397	420	423	16	548
2	0.847	0.093	0.593	0.500	574	398	41	394
1^a^	0.671	0.235	0.759	0.525	735	336	103	233
0	0.430	1	1	0	968	0	439	0

Note: values for sensitivity (ability to correctly identify individuals at risk), specificity (1 — false positives), and case classification at different cut-off points of the Paykel scale are presented. The "Sens-(1-Espec)" column indicates the balance between sensitivity and specificity.

^a^ The optimal cutoff point, identified at 1.0, maximizes sensitivity (75.93%) without excessively compromising specificity (76.54%), providing the best balance for identifying adolescents at high risk of suicide.

## DISCUSSION

 The psychometric analyses conducted with this Spanish version of the PSS in the Mexican context demonstrated a satisfactory fit and high factor loadings. The analysis of the items and the factorial invariance demonstrated the validity of the risk levels in the five questions, regardless of sex at birth, gender identity, or the student’s school grade. Therefore, it can be assumed that this version of the scale is able to efficiently discriminate the adolescent population at risk of SB. Similarly, the results of the study indicated that there is a divergent validity with the DASS-21 scale. Finally, it was determined that an optimal cut-off point of ≥ 1.0 of the PSS accurately identifies adolescents at risk of SB. 

 The results of this study align with previous research on a Latino and Spanish adolescent population, which has also demonstrated the PSS’s adequate psychometric properties^12,15^. It is important to highlight that the robustness of the analyses performed in this study allows us to assert that the PSS is a useful tool for assessing the risk of SB in Mexican adolescent students. 

 According to the latest research, SB is defined as a set of thoughts and behaviors that involve imagining situations where a person could potentially cause harm to themselves or deliberately self-injure with the intention of taking their own life. These behaviors are collectively referred to as suicide ideation, planning, and attempt^33^. Despite the extensive history of research on SB, there remains a degree of controversy and disagreement surrounding the precise definitions of its components^9,21^. Consequently, in certain studies employing PSS, the term "suicidal ideation" is utilized to denote the severity continuum ranging from optimal health and well-being to death by suicide^12,13,15^. The theoretical framework employed by the Center for Disease Control and Prevention^34^, as well as the findings of our study, indicate that the items encompassed by the PSS align with the continuum of severity delineated in the definition of SB, rather than being limited to suicidal ideation alone. 

 The analyses conducted in this study demonstrate the importance of utilizing the PSS in a school context, irrespective of sex at birth (male and female), gender identity (male, female, other), or school grade. This method has been tested with other samples of adolescent students in similar contexts, with similar results in terms of PSS performance when incorporating these variables and in terms of invariance^11^. We believe that incorporating gender identity into our study is essential for achieving a comprehensive understanding of SB in diverse populations. Previous research has identified a link between gender identity and mental health issues, such as depression during adolescence^35^. 

 In contrast to previous studies, which did not establish a cut-off point^12,21^ or treated each positive response to the items as five degrees of SB risk^13^, our results suggest that a cut-off point ≥ 1.0 is adequate to identify students at risk. These findings support the usefulness of the instrument as an effective tool for identifying adolescents at risk for SB, providing a solid criterion for its implementation in clinical assessments and epidemiological studies. However, it is important to note that scales measuring SB are often influenced by the stigma surrounding this phenomenon and may cause discomfort for adolescent and young participants. The brevity and content of the PSS make it a valuable and cost-effective instrument. Our findings suggest that the PSS could be a viable option for use in school settings with adolescents. The PSS is straightforward to implement, does not necessitate specialized personnel, and can be also utilized in population studies. This facilitates the development of public policies and the design of interventions that high schools could implement in collaboration with mental health services. This approach ensures that adolescents receive adequate and timely support. 

 Our study is not free of limitations; a key aspect to consider when interpreting the results is that the sample was composed of adolescents in a school context, excluding those not attending school or clinical populations with a formal diagnosis of mental disorders. While this methodological decision enhances the external validity of the instrument by enabling its application in population screening contexts and epidemiological studies, it also imposes limitations on internal validity. The absence of clinically diagnosed cases may compromise the precision of the cut-off point identified for the PSS. Future studies should consider incorporating clinical samples, ideally with adolescents in psychiatric treatment, which would facilitate a more robust analysis of the instrument’s predictive capacity in scenarios of greater severity. Similarly, exploring differences in factor structure and criterion validity between clinical and non-clinical samples would be beneficial. This would allow us to assess whether the scale functions differently in populations with different levels of risk. This objective could be accomplished by implementing factorial invariance models to assess whether the factor loadings and the instrument’s structure remain constant across both groups. Alternatively, it could be determined whether significant differences exist that necessitate adjustments to the interpretation. 

 We conclude that the PSS is a brief, simple, consistent, and useful test that yields valid results in the Mexican adolescent student population. We have established an optimal cut-off value ≥ 1.0. The scale can be utilized in educational environments to identify students at risk of SB and facilitate their referral to appropriate mental health services. The PSS will be a valuable asset in the implementation of mental health screenings in Mexican schools. 

## Data Availability

The data are available upon request to the corresponding author.
